# Experimental and in silico evidence suggests vaccines are unlikely to be affected by D614G mutation in SARS-CoV-2 spike protein

**DOI:** 10.1038/s41541-020-00246-8

**Published:** 2020-10-08

**Authors:** Alexander J. McAuley, Michael J. Kuiper, Peter A. Durr, Matthew P. Bruce, Jennifer Barr, Shawn Todd, Gough G. Au, Kim Blasdell, Mary Tachedjian, Sue Lowther, Glenn A. Marsh, Sarah Edwards, Timothy Poole, Rachel Layton, Sarah-Jane Riddell, Trevor W. Drew, Julian D. Druce, Trevor R. F. Smith, Kate E. Broderick, S. S. Vasan

**Affiliations:** 1 Commonwealth Scientific and Industrial Research Organisation, Australian Centre for Disease Preparedness, Geelong, VIC 3219 Australia; 2 Commonwealth Scientific and Industrial Research Organisation, Data61, Docklands, VIC 3008 Australia; 3 Victorian Infectious Diseases Reference Laboratory, The Royal Melbourne Hospital at The Peter Doherty Institute for Infection and Immunity, Melbourne, VIC 3000 Australia; 4 Inovio Pharmaceuticals, 10480 Wateridge Circle, San Diego, CA 92121 USA; 5 Department of Health Sciences, University of York, York, YO10 5DD UK

**Keywords:** SARS-CoV-2, Vaccines, Viral evolution

## Abstract

The ‘D614G’ mutation (Aspartate-to-Glycine change at position 614) of the SARS-CoV-2 spike protein has been speculated to adversely affect the efficacy of most vaccines and countermeasures that target this glycoprotein, necessitating frequent vaccine matching. Virus neutralisation assays were performed using sera from ferrets which received two doses of the INO-4800 COVID-19 vaccine, and Australian virus isolates (VIC01, SA01 and VIC31) which either possess or lack this mutation but are otherwise comparable. Through this approach, supported by biomolecular modelling of this mutation and the commonly-associated P314L mutation in the RNA-dependent RNA polymerase, we have shown that there is no experimental evidence to support this speculation. We additionally demonstrate that the putative elastase cleavage site introduced by the D614G mutation is unlikely to be accessible to proteases.

## Introduction

COVID-19 vaccine candidates primarily target the trimeric ‘spike’ (S) glycoprotein, as this factor enables binding to the ‘angiotensin-converting enzyme 2’ (ACE2) host surface receptors and facilitates virus entry into the cells^1^. Over the last few months, an Aspartate-to-Glycine amino acid change has arisen at position 614 of the S protein (resulting from a single A-to-G nucleotide change at position 23,403 in the Wuhan-Hu-1 reference genome), with G614 variants accounting for 75% of published genome sequences worldwide as of 1 July 2020. This mutation has resulted in a number of articles and preprints postulating that isolates containing this ‘D614G’ mutation have a structural advantage^2^, including as a better substrate to the S1 furin cleavage domain^3^, and are associated with an increase in: (a) transmissibility and viral loads^4^; (b) transduction of human cells^2,5^; (c) pathogenicity and case fatality^6^. These have led to speculation that the efficacy of vaccines and countermeasures which target the S protein could be adversely affected, necessitating frequent vaccine matching.

## Results and discussion

We tested this hypothesis with sera from ferrets immunised with a COVID-19 vaccine candidate that targets the S protein (D614 variant), and used biomolecular modelling to interpret our results. We used Australian isolates which either possess or lack the D614G mutation but are otherwise comparable in S protein sequence and also devoid of significant mutations of consequence within viral proteins responsible for cell binding and entry (as discerned with Geneious Prime 2020.1 software; c.f. Supplementary Table 1). Isolated at the Victorian Infectious Diseases Reference Laboratory^7^, the Australian isolates ‘VIC01’ and ‘SA01’ (which are D614) and ‘VIC31’ (which is G614), were used in standard virus neutralisation assays preformed at the Australian Centre for Disease Preparedness, as described under Methods.

**Fig. 1:**
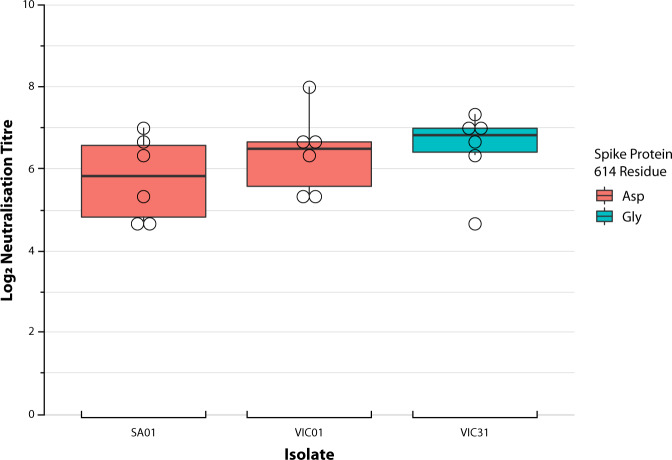
Neutralising titres to three circulating Australian SARS-CoV-2 isolates following prime-boost vaccination with INO-4800. Y-axis represents log_2_ virus neutralising titre to the three Australian SARS-CoV-2 isolates following prime-boost vaccination with INO-4800, including three replicates of each ferret serum sample, represented as a circle for their mean. VIC01 and SA01 virus isolates, possessing a D614, are marked in red; whilst VIC31, possessing a G614, is marked in blue. The dark line on the chart for each virus isolate represents median titre, with the box indicating interquartile range and the vertical line representing the 95% confidence interval. Neutralisation titres against the different virus isolates are not significantly different.

Previous studies in rodents with INO-4800 have demonstrated the induction of humoural and cellular immune responses targeting SARS-CoV-2 spike protein^8^. In this study, ferrets were shown to have developed SARS-CoV-2 neutralising antibody responses following vaccination with INO-4800, demonstrating that ferrets are an appropriate animal to model COVID-19 vaccine immunogenicity, and that this DNA vaccine stimulates an effective B cell response. The overall median log_2_ neutralisation titre against the three virus isolates combined was 6.32 (range 4.32 to 8.32). Comparison of the titres by virus isolate (SA01, VIC01, and VIC31) revealed that the D614G mutation had little effect on neutralisation efficiency following vaccination (Fig. 1). Indeed, the overall log transformed mean neutralisation titres for the VIC31 variant (the G614 variant) were not significantly different than those for the SA01 and VIC01 isolates possessing the D614 (*p* > 0.05).

**Fig. 2:**
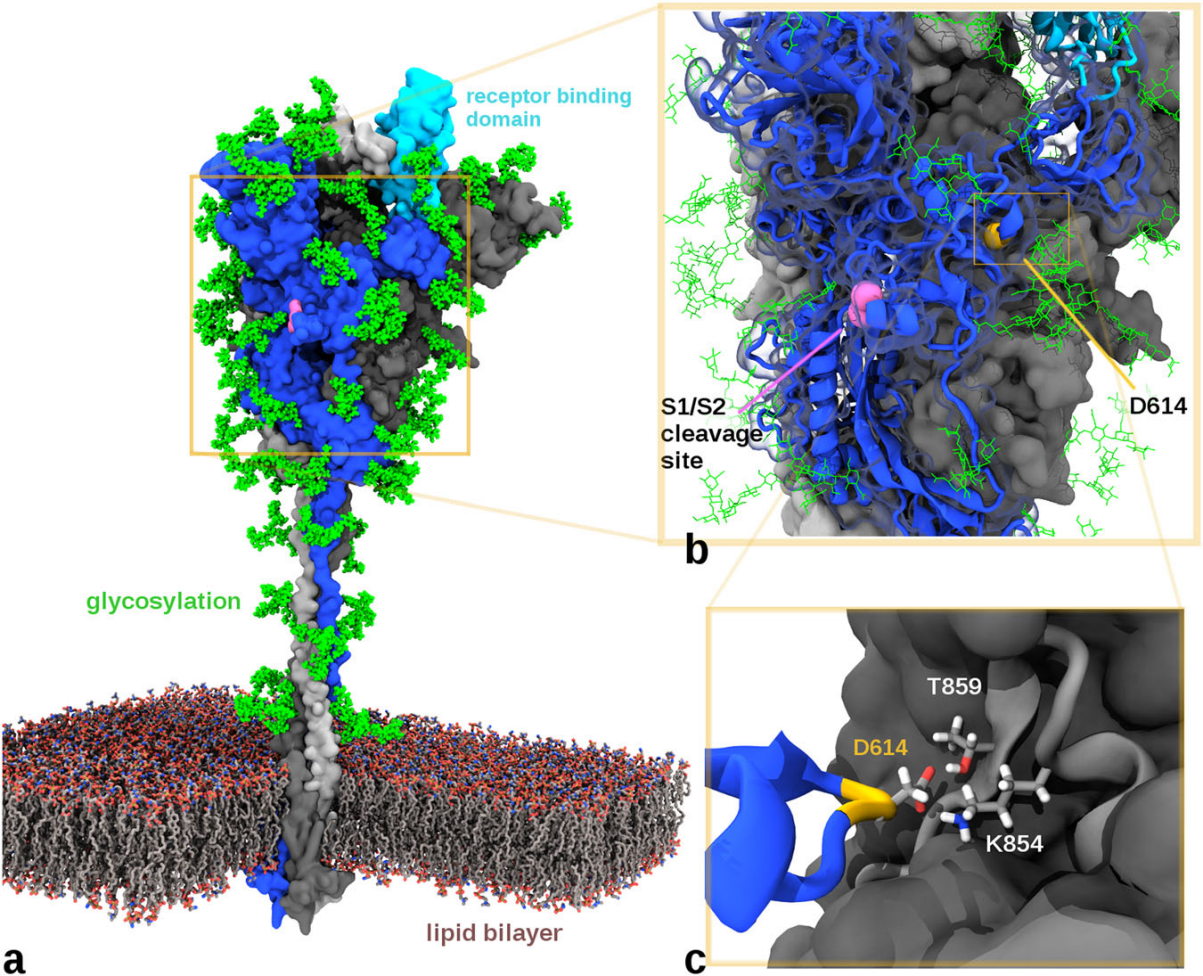
Biomolecular model of SARS-CoV-2 spike protein with location of D614 residue. **a** Model of the glycosylated Spike protein trimer (based on Woo et al.^9^) depicting its insertion through a lipid bilayer and the relative position of a receptor binding domain in an ‘up’ position of the blue/cyan chain (other S chains coloured light and dark grey). **b** Close-up depicting the positioning of the solvent-accessible S1/S2 cleavage site (residues R685-S686) as well as the recessed and glycan-protected position of the D614 residue. **c** Cut-away view of the D614 interface with the adjacent S chain in grey. D614 can hydrogen bond with T859 and form a salt bridge with K854. A D614G mutation is expected to disrupt both these interactions.

We used molecular models of the spike protein (both our molecular dynamics simulations and another spike model^9^) to examine the structural context of the D614G mutation to address possible concerns of adverse vaccine efficacy and the plausibility of recently-proposed selection advantages^2–5^. The S protein of coronavirus comprises of two sections, S1 and S2, and forms a heavily glycosylated transmembrane trimer facilitating both host attachment (via the receptor binding domain ‘RBD’ in S1), and cell fusion/entry (via a trimeric membrane fusion S2 stalk) after proteolytic cleavage of the S1/S2 junction^10^ (Fig. 2a). The SARS-CoV-2 S protein is different to related coronaviruses as it contains a proprotein convertase (PPC) motif at the S1/S2 boundary, which has been shown to be pre-cleaved in pseudovirus cellular entry assays by the proprotein convertase furin^10,11^.

The 614 position in S is upstream of the S1/S2 furin cleavage site (R685/S686)^9^ and is recessed at a buried interface position of the adjacent monomer while additionally shielded by an N-linked glycan at position N616^12^ (Fig. 2b). Given its internal position, the G614 variant is unlikely to be a component of neutralising epitopes on the S protein and thus a lack of adverse effects on neutralisation efficiency of antibodies generated following vaccination with D614-derived vaccines is not unexpected. Indeed, the location of residue 614 in S is distinct from both of the linear neutralising epitopes^13^.

Whilst we agree with studies suggesting that the D614G mutation introduces an elastase cleavage site^14^, our molecular dynamics simulations do not support their inference of increased replication efficiency by promoting elastase-mediated S1/S2 cleavage. Modelling of S in the presence of both furin and elastase proteases suggest that, while furin can access the S1/S2 cleavage site (Fig. S1), elastase is unable to access the putative G614-introduced elastase cleavage site due to its interface positioning and glycan blocking (Fig. S2a) unless the S1 trimer cap and S2 separate (Fig. S2b).

Recent findings^2^ suggested the superior infectivity of G614 is through the D614G mutation stabilising the interaction between the S1 and S2 domains. However, our examination of D614 (located in S1) reveals interactions with S2, not only through hydrogen bond interactions with an adjacent Thr859 as previously noted^4^, but also a salt bridge with residue Lys854 (Fig. 2c). The aspartate-to-glycine change at 614 removes the inter-chain D614-K854 and might actually destabilise the interaction between S1 and S2 domains.

Our in silico approach has shed additional insights into a couple of mechanisms that warrant further investigation, including experiments. Firstly, although our simulation considered interactions between human ACE2 and the RBD in the traditional ‘up’ orientation, over the course of a short 100-ns simulation we observed the ACE2 tilt and contact the adjacent RBD via V445 making close contact with P321 and F555 on ACE2. This suggests that the adjacent RBD in the ‘down’ conformation could contribute to ACE2 binding and specificity (Fig. S3), a hypothesis supported by previous work^15^ highlighting structural flexibility with three distinct conformations of SARS-CoV S protein in complex with ACE2.

Secondly, a parallel C-to-U substitution at position 14,408 in the genome, resulting in a P323L (P314L in orf1b) mutation in the RNA-dependent polymerase (RdRp/nsp12), has been associated with D614G^16^ (including in VIC31) and should therefore be considered for potential contribution to infectivity. The P323 position is in a hydrophobic cleft approximately 30 Å from the catalytic site of RdRp, but close to the nsp8 interface (Fig. S4). It is not immediately clear what effect the P323L mutation has on virulence. The Pro323 to Leu mutation may relieve some backbone constraints and contribute local conformational stability. We noted in our model based on pdb structure 6YYT^17^ that the cleft in nsp12 is predominately lined with arginine residues (R173, R249, R349 & R457) which may contribute to nucleotide binding due to the strong association with arginine and phosphate groups^18^ (Fig. S4). The P323L mutation appears to partially obstruct R349 in the cleft. We are yet to establish the significance of this mutation.

Given the rapidity with which this virus has emerged, correlates of protection have yet to be established for immune responses. Ideally, passive-protection assays would also be performed in future studies to determine in vivo effects. The experimental and biomolecular modelling approaches described above are available at many organisations around the world such as ours. Therefore, it would be desirable to analyse the impact of identified mutations, in collaboration with such organisations, before speculating on potential adverse effects on vaccines. As infectious clones are established^19–21^, it will be easier to study accumulated mutations, which are inevitable for this RNA virus, even with an exoribonuclease ‘proof-reading’ capacity. Therefore, we urge caution when these mutations are described in preprint articles^2–5^ because premature inferences on their effects without supporting experimental evidence could result in a media frenzy and potentially undermine public confidence in vaccines.

## Methods

### Viruses, vaccine, and cells

Australian SARS-CoV-2 isolates hCoV-19/Australia/VIC01/2020 (VIC01), hCoV-19/Australia/SA01/2020 (SA01), and hCoV-19/Australia/VIC31/2020 (VIC31) were provided as Passage 1 material by the Victorian Infectious Diseases Reference Laboratory (VIDRL), with the support of South Australia Pathology (SA01 isolate)^7^.

INO-4800 was provided by Inovio Pharmaceuticals for preclinical testing within the ferret model of COVID-19. This DNA vaccine is a plasmid containing a coding sequence for SARS-CoV-2 (Wuhan-Hu-1) Spike glycoprotein without constrains on protein folding^8^.

VIC01 Passage 2 virus stock was grown in VeroE6 cells from BEI Resources (Manassas, VA, USA), whilst SA01 and VIC31 Passage 2 virus stocks were grown in VeroE6 cells (European Collection of Animal Cell Cultures; Porton Down, UK). Briefly, VeroE6 cells were grown in 150 cm^2^ flasks in Dulbecco’s Modified Eagle Medium (DMEM) containing 10% heat-inactivated foetal bovine serum (FBS), 10 mM HEPES, 100 U/mL penicillin, 100 μg/mL streptomycin, and 250 ng/mL amphotericin B (all components from ThermoFisher Scientific, Scoresby, VIC, Australia) until 79–90% confluent. The Passage 1 material for each SARS-CoV-2 isolate received from VIDRL was diluted 1:100 in PBS (VIC01) or DMEM containing 10 mM HEPES, 100 U/mL penicillin, 100 μg/mL streptomycin, and 250 ng/mL amphotericin B, but no FBS (DMEM-D; SA01 and VIC31). Cells were inoculated with 2 mL (VIC01) or 4 mL (SA01 and VIC31) diluted virus and were incubated for 30 min at 37 °C/5% CO_2_ before 40 mL DMEM containing 2% FBS, 10 mM HEPES, 100 U/mL penicillin, 100 μg/mL streptomycin, and 250 ng/mL amphotericin B was added. The flasks were incubated for an additional 48–72 h before supernatant was harvested. Each of the virus stocks were confirmed to be free from mycoplasma contamination. ECACC VeroE6 cells were additionally used for virus neutralisation assays (see below).

### Vaccination and serum samples

Four male and four female ferrets received two doses of INO-4800 via intramuscular administration of 1 mg plasmid DNA to the caudal thigh muscle, followed by electroporation split across two sites using the CELLECTRA® device. The prime dose was given on day 0 and the boost on day 28. Sera collected on day 35 or day 42 from three male and three female ferrets were chosen at random to allow for sufficient seroconversion, and to ensure that no ferret provided more than one test sample (c.f. Supplementary Table 2). Three unvaccinated ferret sera and a hyperimmune horse anti-SARS-CoV serum sample were also included as positive and negative control sera, respectively, to ensure that the assay was performing appropriately. Vaccine efficacy studies were approved by the Animal Ethics Committee at CSIRO ACDP.

### Serum dilution

Each serum sample was diluted 1:20 in DMEM-D (see cell culture methods above) in a deep-well plate on a single occasion, followed by a twofold serial dilution in medium across the plate up to 1:20,480. The dilution series for each serum sample was dispensed into triplicate rows of a 96-well plate, for a total volume of 50 μL per well and triplicate wells per sample dilution.

### Neutralisation assay

For the serum-containing wells, 50 μL virus diluted in medium to contain approximately 100 TCID_50_ (confirmed by back-titration) was added to each well. The plates were incubated at 37 °C/5% CO_2_ for 1 hour to allow neutralisation complexes to form between the antibodies and the virus. At the end of the incubation, 100 μL VeroE6 cells (propagated as outlined above for virus stock generation) were added to each well and the plates returned to the incubator for 4 days. Each well was scored for the presence of viral CPE, readily discernible on Day 4 post-infection, with neutralisation titres assigned to each serum replicate based upon the highest dilution that prevented discernible cytopathic effect.

### Statistical analysis

Mixed effects analysis of variance (ANOVA) was used to assess differences between SARS-CoV-2 isolates (the fixed effect), with the random effect being the 3 test replicates. For the post hoc analysis to detect significant factor level differences we undertook pairwise comparisons with Tukey’s adjustment. All analyses were undertaken in R 4.0, using the *nlme v. 3.1*. package for the mixed effects ANOVA modelling and *multcomp v. 1.4* for the post hoc comparisons.

### Molecular modelling

Fully glycosylated models of the S protein wildtype (D614) and variant (G614) were built based on ‘6VSB’ protein databank (PDB) structure^1^ minus transmembrane domains. The G614 model had additional ACE2, elastase and furin proteins (based on pdb structures 6M17, 4WVP and 5JXG respectively) to assess their accessibility to their respective binding and proteolytic cleavage sites. An additional solvated model of RdRp was built to help assess the P323L mutation. All models were simulated in aqueous solution (TIP3 water, 310 K, 0.15 M ions, NVT ensemble) using the software NAMD 2.13^22^. Models were visualised with VMD and Nanome^23–25^ (more details in Supplementary Material).

### Approval for animal studies

All relevant ethical regulations for animal testing and research were adhered to. The parent vaccine efficacy study was reviewed and approved by the Animal Ethics Committee (AEC) of the CSIRO Australian Centre for Disease Preparedness (Approval Reference: AEC 2004). No additional approval was required to perform neutralisation assays on serum samples collected from the parent study.

### Reporting summary

Further information on research design is available in the Nature Research Reporting Summary linked to this article.

## Supplementary information

Supplemental Material

Supplementary Video 1

Reporting Summary

## Data Availability

Raw neutralisation titres used for the preparation of Fig. 1 can be found in Supplementary Materials. Biomolecular modelling was performed using publicly-available structures cited in the manuscript.
