# Supplementary material for: Experimental and in silico evidence suggests vaccines are unlikely to be affected by D614G mutation in SARS-CoV-2 spike protein
Source: NPJ Vaccines. 2020 Oct 8;5:96. doi: 10.1038/s41541-020-00246-8 (PMC7546614; doi:10.1038/s41541-020-00246-8)
Supplement: Supplementary file 1 — Supplemental Material [file 41541_2020_246_MOESM1_ESM.pdf]

Experimental and *in silico* Evidence Suggests Vaccines are Unlikely to be Affected by D614G Mutation in SARS-CoV-2 Spike Protein

Supplementary Material

Alexander J. McAuley[1], Michael J. Kuiper[2], Peter A. Durr[1], Matthew P. Bruce[1], Jennifer Barr[1], Shawn Todd[1], Gough G. Au[1], Kim Blasdell[1], Mary Tachedjian[1], Sue Lowther[1], Glenn A. Marsh[1], Sarah Edwards[1], Timothy Poole[1], Rachel Layton[1], Sarah-Jane Riddell[1], Trevor W. Drew[1], Julian D. Druce[3], Trevor R.F. Smith[4], Kate E. Broderick[4] & S.S. Vasan[1,5*]

[1] Commonwealth Scientific and Industrial Research Organisation, Australian Centre for Disease Preparedness, Geelong, VIC 3219, Australia
[2] Commonwealth Scientific and Industrial Research Organisation, Data61, Docklands, VIC 3008, Australia
[3] Victorian Infectious Diseases Reference Laboratory, The Royal Melbourne Hospital at The Peter Doherty Institute for Infection and Immunity, Melbourne, VIC 3000, Australia
[4] Inovio Pharmaceuticals, 10480 Wateridge Circle, San Diego, California, 92121, USA
[5] University of York, Department of Health Sciences, York, YO10 5DD, United Kingdom

Supplementary Figures

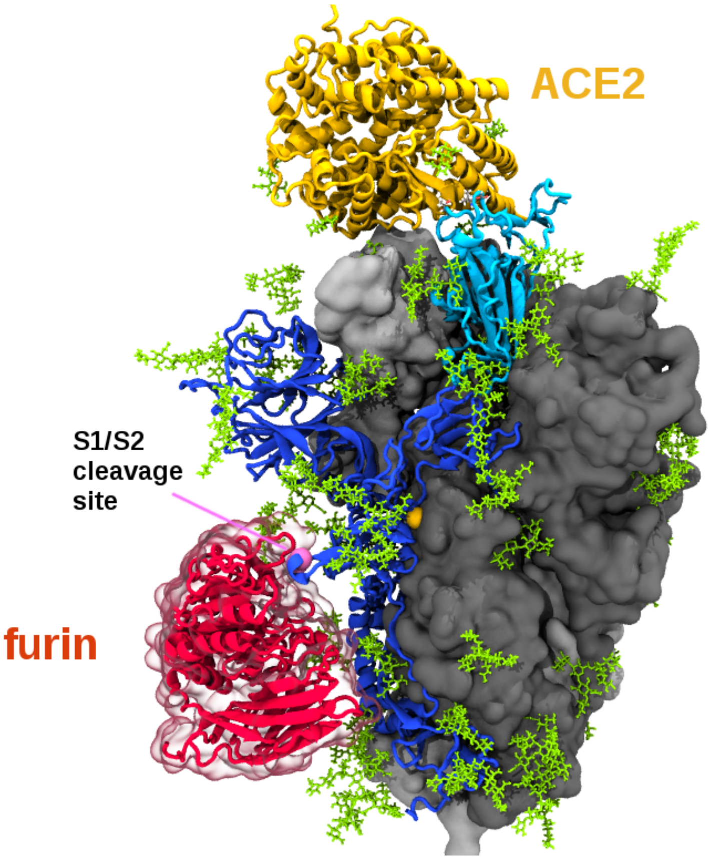

Supplementary Figure 1. Snapshot of unconstrained molecular dynamics simulation showing proprotein convertase (PPC) furin (red) accessibility of the solvent-exposed S1/S2 cleavage site. It also depicts the binding of ACE2 (yellow) binding to the receptor binding domain (Cyan). Glycosylation is shown in green.

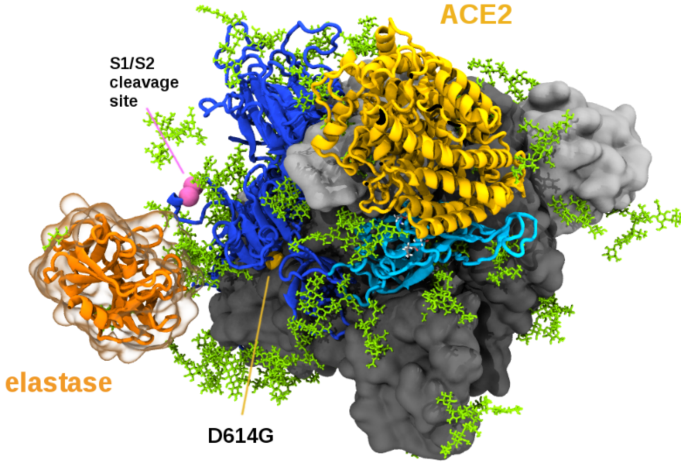

a.

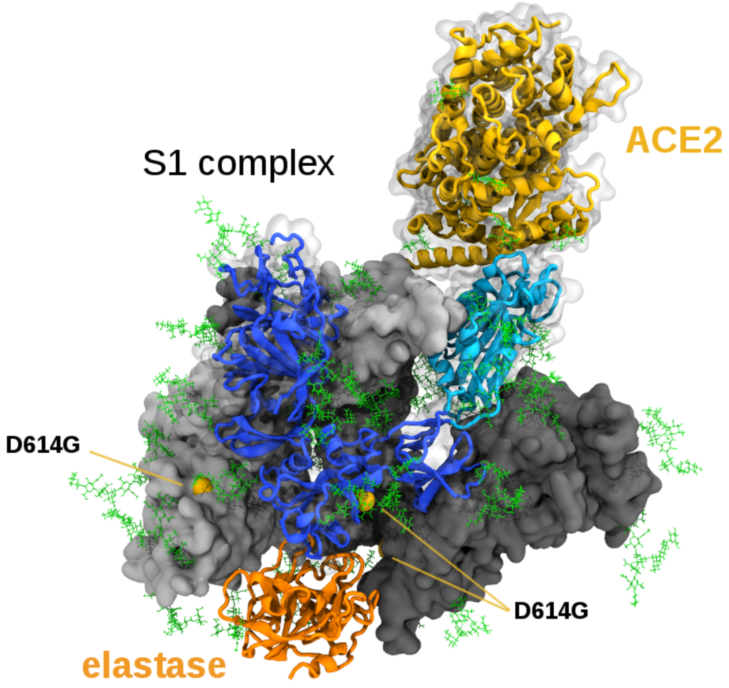

b.

Supplementary Figure 2a**).** Top view of an unconstrained molecular dynamics simulation showing neutrophil elastase (orange) unable to access the putative elastase cleavage site created by the D614G mutation due to glycan shielding particularly from N-linked N616.
b). A cleaved S1 complex modelled with elastase demonstrating accessibility to the 614 site. Both figures also depict the binding of ACE2 (yellow) binding to the receptor binding domain (Cyan). Glycosylation is shown in green.

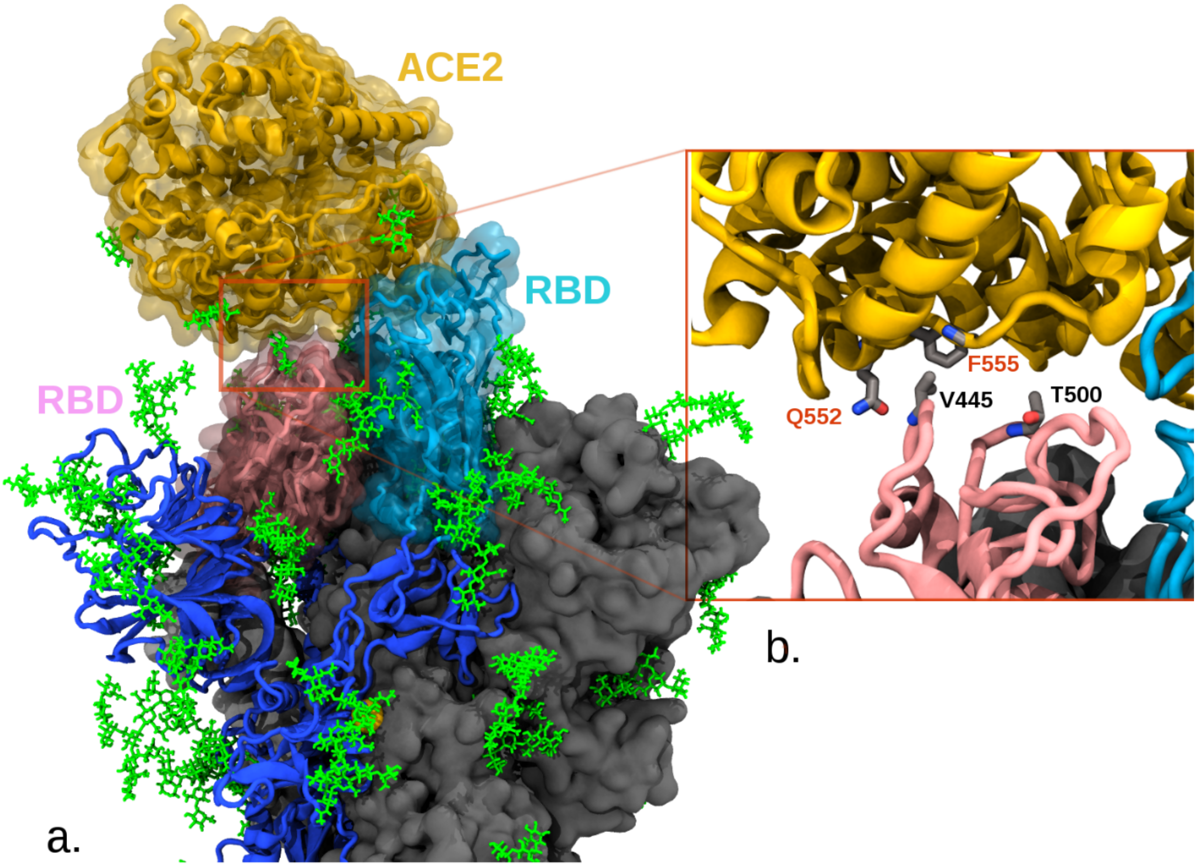

Supplementary Figure 3.a) Model of the ACE2 (yellow) interacting with two receptor binding domains (RBD), one in the 'up' conformation (cyan) and the adjacent RBD (pink) in the 'down' conformation. b). Close-up of the ACE2 and RBD 'down' showing proximity of V445 and T500 of RBD with ACE2 Q552 & F555.

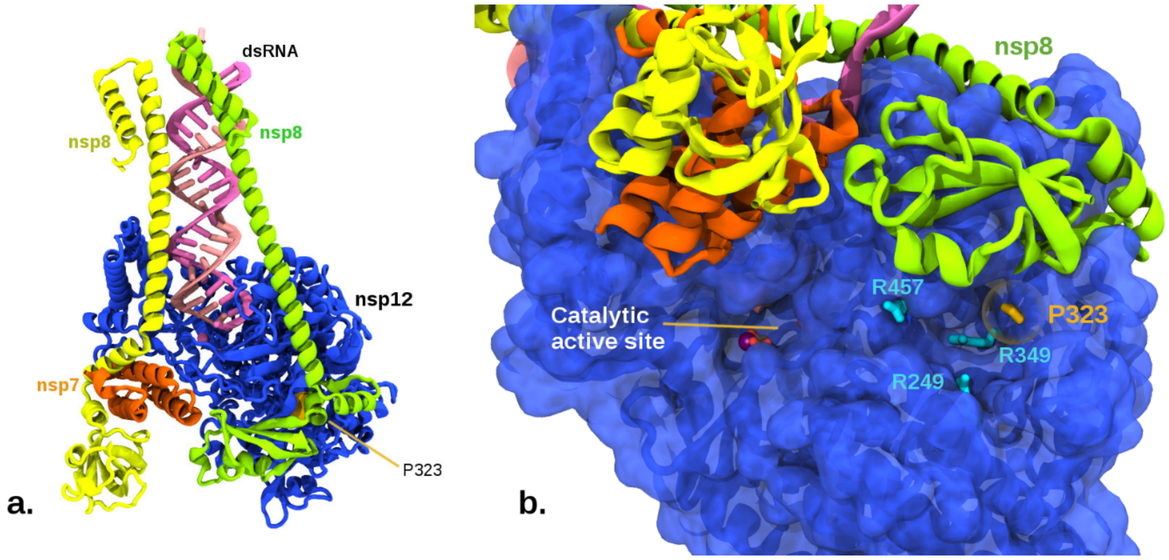

Supplementary Figure 4. a) Model of the COVID 19 RNA-dependant RNA polymerase (RdRp/nsp12 (blue)) with double stranded RNA (pink and mauve) and complexed nsp7/nsp8 (orange, yellow and green) based on pdb structure 6YYT[17]; b) position P323 is approximately 30 Å from the catalytic active site, but adjacent to an nsp8 interface shown in green.

Nanome Virtually Reality File:
Virtual reality model of Spike protein.
This file will allow users with a VR headset and Nanome installed to visually inspect and manipulate the spike model.
https://www.dropbox.com/s/on5ophmwjug833i/nanome_Spike_model.nanome?dl=0
Nanome VR can be freely downloaded from www.nanome.ai.

Supplementary Movie
https://www.dropbox.com/s/8ihzisj09xa2ydf/AEF_sim_010001-0565.mp4?dl=0
Molecular dynamics simulation of SARS-CoV-2 spike protein investigating accessibility of furin and elastase enzymes to putative proteolytic sites.

Supplementary Tables

Supplementary Table 1: Mutations in Australian isolates compared to Wuhan-Hu-1
This list indicates that the Spike region is comparable between the chosen isolates, which are also devoid of significant mutations of consequence within viral proteins responsible for cell binding and entry. Mutations in the Spike protein are highlighted in **Bold**
Source: GISAID (https://www.gisaid.org/)

Isolate	Mutation	Consequence
VIC01	U19065C	Silent
	**U22303G**	**S247R in Spike protein**
	G26144U	G521V in ORF3a protein
	29750-29759 Deletion	Deletion in 3’ untranslated region
SA01	C3037U	Silent
	C17074U	L279F in Helicase peptide / L1203F in orf1b
	26284-26286 Deletion	Loss of V14 in E protein
	C27213U	Silent
	U27384C	Silent
VIC31	C241U	Mutation in 5’ untranslated region
	C1059T	T85I in nsp2 peptide
	C3037U	Silent
	C14408U	P323L in RdRP protein / P314L in orf1b
	**A23403G**	**D614G in Spike protein**
	G25563U	Q57H in ORF3a protein

Supplementary Table 2: Vaccination groups

Eight ferrets (4 male, 4 female) were administered prime and boost doses of INO-4800. Of these, we selected three ferrets of each sex at random for use in neutralisation assays as a balance between statistical power and preservation of sample availability for ongoing vaccine studies.

Vaccine	Route of Administration	Dose
Inovio (INO-4800)	Intramuscular injection followed by electroporation using CELLECTRA® device	1mg pGX9501 plasmid DNA

Supplementary Table 3: Neutralisation titres

Log2 neutralisation titres (technical replicates) for individual ferrets against SARS-CoV-2 SA01, VIC01, and VIC31 isolates following INO-4800 prime (Day 0) and boost (Day 28) vaccination. Study day of sample collection and animal sex also listed.

			**Triplicate Neutralisation Titres (Log2)**		
**Ferret Number**	**Sex**	**Study Day**	**SA01**	**VIC01**	**VIC31**
59	M	35	7.32, 6.32, 7.32	8.32, 7.32, 8.32	7.32, 6.32, 7.32
62	M	35	7.32, 6.32, 6.32	6.32, 6.32, 7.32	7.32, 6.32, 6.32
67	M	42	4.32, 4.32, 5.32	5.32, 5.32, 5.32	6.32, 6.32, 6.32
76	F	35	5.32, 5.32, 5.32	6.32, 6.32, 6.32	7.32, 6.32, 7.32
70	F	35	6.32, 6.32, 6.32	7.32, 6.32, 6.32	7.32, 7.32, 7.32
82	F	42	4.32, 5.32, 4.32	5.32, 5.32, 5.32	5.32, 4.32, 4.32
		**Median**	5.82	6.32	6.32

Supplementary Methods

Molecular modelling.

Molecular simulations were performed using NAMD2.13[1] with CHARM36m[2] forcefield employing a TIP3 water model. The Spike model was based on the pdb structure 6VSB[3]. Glycosylation of the spike protein was manually constructed using VMD guided by glycan analysis[4].

Simulations were run with Periodic Boundary Conditions 'PBCs' using the NPT ensemble at 310K and 1 bar pressure employing Langevin dynamics. The PBCs were constant in the XY dimensions. Long-range Coulomb forces were computed with the Particle Mesh Ewald

method with a grid spacing of 1 Å. 2 fs timesteps were used with non-bonded interactions calculated every 2 fs and full electrostatics every 4 fs while hydrogens were constrained with the SHAKE algorithm. The cut-off distance was 12 Å with a switching distance of 10 Å and a pair-list distance of 14 Å. Pressure was controlled to 1 atmosphere using the Nosé-Hoover Langevin piston method employing a piston period of 100 fs and a piston decay of 50 fs. Trajectory frames were captured every 100 ps.

Our main model construct included truncated Spike protein complex (residues 27-1147 with D614G mutation), ACE2 (based on pdb:6M17), elastase (pdb: 4WVP), and furin (pdb: 5JXG). Each additional protein was positioned deliberately close to their respective putative sites of interactions on the spike. This was solvated to dimensions 195x190x250Å and ionized to approximately 0.15M (579 Na^+, 60 K^+, 10 Mg^{2+} , 633 Cl^-) for a total of 816,161 atoms. This simulation was run for 150 nanoseconds without constraints. Trajectories were visually examined for proximity and conformational changes. Furin was observed to make direct contact with the furin cleavage site (R685/S686), while elastase was obstructed from G614 by glycosylation, never approaching closer than 27 Å. ACE2 bound to the receptor binding domain (RBD) as expected, however was observed to tilt and make contact to the adjacent RBD via V445 and T500. The contact between ACE2 and the second RBD appears to desolvate the interface.

A second model which included the cleaved S1 complex with ACE2 and elastase was constructed to test accessibility of the latter to the G614 site. The S1 complex was based on residues 1 to 685[12]. This was solvated to dimensions 180x190x150Å and ionized to approximately 0.15M (355 Na^+, 50 K^+, 10 Mg^{2+} , 426 Cl^-) for a total of 505,236 atoms. This simulation was run for 70 ns without constraints, with the elastase initially positioned near the cavity left by the S2 domain. A weak distance constraint was applied for 2 ns to nudge the elastase towards the centre of the cavity before removing all constraints and simulating a further 26 ns. The 26.5kDa elastase appeared small enough to fit the S1 cavity and approach the 614 site from the inside.

Our third model of the RNA polymerase complex was based on pdb structure 6YYT, consisting of SARS CoV-2 protein structures (nsp12, two nsp8, and nsp7) as well as a complementary double stranded RNA model. This was solvated to dimensions 140x120x160Å and ionized to approximately 0.15M (20 Na^+, 189 K^+, 52 Mg^{2+} ,227 Cl^-) for a total of approximately 266,000 atoms. Both P323 and L323 variants were constructed and run for 50 ns each. No significant structural changes were noted with the two variants.

1 Phillips, J. C. *et al.* Scalable molecular dynamics with NAMD. *J Comput Chem* **26**, 1781-1802, doi:10.1002/jcc.20289 (2005).

2 Huang, J. *et al.* CHARMM36m: an improved force field for folded and intrinsically disordered proteins. *Nature Methods* **14**, 71-73, doi:10.1038/nmeth.4067 (2017).

3 Wrapp, D. *et al.* Cryo-EM structure of the 2019-nCoV spike in the prefusion conformation. *Science* **367**, 1260-1263 (2020).

4 Watanabe, Y., Allen, J. D., Wrapp, D., McLellan, J. S. & Crispin, M. Site-specific glycan analysis of the SARS-CoV-2 spike [ePublish Ahead of Print]. *Science* (2020).
