# Supplementary material for: Experimental and in silico evidence suggests vaccines are unlikely to be affected by D614G mutation in SARS-CoV-2 spike protein
Source: NPJ Vaccines. 2020 Oct 8;5:96. doi: 10.1038/s41541-020-00246-8 (PMC7546614; doi:10.1038/s41541-020-00246-8)
Supplement: Supplementary file 3 — Reporting Summary [file 41541_2020_246_MOESM3_ESM.pdf]

nature research

Corresponding author(s): Seshadri Vasan
Last updated by author(s): Sep 18, 2020

Reporting Summary

Nature Research wishes to improve the reproducibility of the work that we publish. This form provides structure for consistency and transparency in reporting. For further information on Nature Research policies, see our Editorial Policies and the Editorial Policy Checklist.

Statistics

For all statistical analyses, confirm that the following items are present in the figure legend, table legend, main text, or Methods section.

n/a	Confirmed	
☐	☒	The exact sample size (n) for each experimental group/condition, given as a discrete number and unit of measurement
☐	☒	A statement on whether measurements were taken from distinct samples or whether the same sample was measured repeatedly
☐	☒	The statistical test(s) used AND whether they are one- or two-sided<br>*Only common tests should be described solely by name; describe more complex techniques in the Methods section.*
☐	☒	A description of all covariates tested
☐	☒	A description of any assumptions or corrections, such as tests of normality and adjustment for multiple comparisons
☐	☒	A full description of the statistical parameters including central tendency (e.g. means) or other basic estimates (e.g. regression coefficient) AND variation (e.g. standard deviation) or associated estimates of uncertainty (e.g. confidence intervals)
☐	☒	For null hypothesis testing, the test statistic (e.g. F, t, r) with confidence intervals, effect sizes, degrees of freedom and P value noted<br>*Give P values as exact values whenever suitable.*
☒	☐	For Bayesian analysis, information on the choice of priors and Markov chain Monte Carlo settings
☒	☐	For hierarchical and complex designs, identification of the appropriate level for tests and full reporting of outcomes
☒	☐	Estimates of effect sizes (e.g. Cohen's d, Pearson's r), indicating how they were calculated

Our web collection on statistics for biologists contains articles on many of the points above.

Software and code

Policy information about availability of computer code

Data collection	No software was used
Data analysis	R 4.0 using nlme v3.1 and multcomp v1.4 for statistical analysis. NAMD2.13, VMD, and Nanome for biomolecular modelling

For manuscripts utilizing custom algorithms or software that are central to the research but not yet described in published literature, software must be made available to editors and reviewers. We strongly encourage code deposition in a community repository (e.g. GitHub). See the Nature Research guidelines for submitting code & software for further information.

Data

Policy information about availability of data

All manuscripts must include a data availability statement. This statement should provide the following information, where applicable:

- Accession codes, unique identifiers, or web links for publicly available datasets
- A list of figures that have associated raw data
- A description of any restrictions on data availability

All data are available

Field-specific reporting

Please select the one below that is the best fit for your research. If you are not sure, read the appropriate sections before making your selection.

☒ Life sciences ☐ Behavioural & social sciences ☐ Ecological, evolutionary & environmental sciences

For a reference copy of the document with all sections, see nature.com/documents/nr-reporting-summary-flat.pdf

Life sciences study design

All studies must disclose on these points even when the disclosure is negative.

Sample size	Eight ferrets were vaccinated with INO-4800. Of these, six samples were selected at random for analysis
Data exclusions	No data excluded
Replication	Six biological replicates were used for the study. Three technical replicates were used in the neutralisation study for each serum/isolate combination
Randomization	Randomisation was performed for the animal assignment for the parent vaccine study, but was not applicable for the study described in this manuscript
Blinding	Blinding was performed during the parent vaccine study, but was not applicable for the study described in this manuscript

Reporting for specific materials, systems and methods

We require information from authors about some types of materials, experimental systems and methods used in many studies. Here, indicate whether each material, system or method listed is relevant to your study. If you are not sure if a list item applies to your research, read the appropriate section before selecting a response.

Materials & experimental systems

n/a	Involved in the study	
☒	☐	Antibodies
☐	☒	Eukaryotic cell lines
☒	☐	Palaeontology and archaeology
☐	☒	Animals and other organisms
☒	☐	Human research participants
☒	☐	Clinical data
☒	☐	Dual use research of concern

Methods

n/a	Involved in the study	
☒	☐	ChIP-seq
☒	☐	Flow cytometry
☒	☐	MRI-based neuroimaging

Eukaryotic cell lines

Policy information about cell lines

Cell line source(s)	ECACC
Authentication	Cells were originally authenticated by ECACC and used within 20 passages of previous authentication
Mycoplasma contamination	Cells were negative for mycoplasma (tested by qPCR)
Commonly misidentified lines (See ICLAC register)	N/A

Animals and other organisms

Policy information about studies involving animals; ARRIVE guidelines recommended for reporting animal research

Laboratory animals	Samples collected from ferrets as part of vaccine efficacy testing. Samples used in this study were from three male and three female animals
Wild animals	N/A
Field-collected samples	N/A

Ethics oversight	CSIRO ACDP Animal Ethics Committee

Note that full information on the approval of the study protocol must also be provided in the manuscript.
